# Accelerating Complete Phytoplasma Genome Assembly by Immunoprecipitation-Based Enrichment and MinION-Based DNA Sequencing for Comparative Analyses

**DOI:** 10.3389/fmicb.2021.766221

**Published:** 2021-11-11

**Authors:** Choon Meng Tan, Yu-Chen Lin, Jian-Rong Li, Yuan-Yu Chien, Chien-Jui Wang, Lin Chou, Cheng-Wei Wang, Yi-Ching Chiu, Chih-Horng Kuo, Jun-Yi Yang

**Affiliations:** ^1^Institute of Biochemistry, National Chung Hsing University, Taichung, Taiwan; ^2^Institute of Plant and Microbial Biology, Academia Sinica, Taipei, Taiwan; ^3^Institute of Genomics and Bioinformatics, National Chung Hsing University, Taichung, Taiwan; ^4^Ph.D. Program in Microbial Genomics, National Chung Hsing University and Academia Sinica, Taichung, Taiwan; ^5^Institute of Biotechnology, National Chung Hsing University, Taichung, Taiwan; ^6^Advanced Plant Biotechnology Center, National Chung Hsing University, Taichung, Taiwan

**Keywords:** phytoplasma, Nanopore, effector, SAP11, genome sequencing, potential mobile unit

## Abstract

Phytoplasmas are uncultivated plant-pathogenic bacteria with agricultural importance. Those belonging to the 16SrII group, represented by ‘*Candidatus* P. aurantifolia’, have a wide range of plant hosts and cause significant yield losses in valuable crops, such as pear, sweet potato, peanut, and soybean. In this study, a method that combines immunoprecipitation-based enrichment and MinION long-read DNA sequencing was developed to solve the challenge of phytoplasma genome studies. This approach produced long reads with high mapping rates and high genomic coverage that can be combined with Illumina reads to produce complete genome assemblies with high accuracy. We applied this method to strain NCHU2014 and determined its complete genome sequence, which consists of one circular chromosome with 635,584 bp and one plasmid with 4,224 bp. Although ‘*Ca*. P. aurantifolia’ NCHU2014 has a small chromosome with only 471 protein-coding genes, it contains 33 transporter genes and 27 putative effector genes, which may contribute to obtaining nutrients from hosts and manipulating host developments for their survival and multiplication. Two effectors, the homologs of SAP11 and SAP54/PHYL1 identified in ‘*Ca*. P. aurantifolia’ NCHU2014, have the biochemical activities in destabilizing host transcription factors, which can explain the disease symptoms observed in infected plants. Taken together, this study provides the first complete genome available for the 16SrII phytoplasmas and contributes to the understanding of phytoplasma pathogenicity.

## Introduction

Phytoplasmas are wall-less bacterial pathogens that are known to infect numerous plant species and lead to significant agricultural losses (Gurr et al., 2016; Kumari et al., 2019; Pierro et al., 2019). They are parasitic bacteria multiplying exclusively in phloem sieve elements and are transmitted between plants by phloem-feeding insects (Lee et al., 2000; Hogenhout et al., 2008). Plants infected by phytoplasmas exhibit a range of symptoms, including witches’ broom, phyllody, virescence, purple top, stunting, yellowing, and general decline (Christensen et al., 2005). To date, more than 40 *Candidatus* Phytoplasma (abbreviated as *Ca*. P.) species have been described (Kumari et al., 2019). However, as an obligate intracellular parasite, phytoplasmas remain some of the most challenging plant pathogens to characterize due to the lack of an axenic culture.

Beginning with the important step in whole-genome sequencing of phytoplasmas, many aspects have been intensively studied, and a better understanding of the molecular interactions between phytoplasmas and their hosts is revealed (Oshima et al., 2004; Namba, 2019). Overall, phytoplasmas have relatively small genomes (i.e., < 1,000 kb) compared with other bacteria, and many metabolic genes required for biosynthetic pathways of indispensable compounds are missing, including those for the biosynthesis of amino acids, nucleotides and fatty acids, the tricarboxylic acid cycle, and ATP synthases (Oshima et al., 2004, 2013; Namba, 2019). As a result, phytoplasmas rely on an enriched environment and their transporters to obtain nutrients from hosts for their survival and multiplication. At the same time, pathogenicity factors (i.e., effectors), such as TENGU, SAP11, and SAP54/PHYL1, are secreted by phytoplasmas to interfere with plant developmental processes as well as plant hormone homeostasis (Hoshi et al., 2009; Sugio et al., 2011a; MacLean et al., 2014; Maejima et al., 2014; Tan et al., 2016; Chang et al., 2018). The morphological and physiological changes of host plants not only associate with symptom development, but also improve the fitness of phytoplasmas and their insect vectors, resulting in the spread of phytoplasma diseases (Tomkins et al., 2018; Dermastia, 2019).

The spreading of phytoplasmas might also rely on the interactions between phytoplasma immunodominant membrane proteins (IDPs) and host proteins (Konnerth et al., 2016). IDPs are highly abundant proteins in the cell membrane of phytoplasmas, which can be grouped into three classes, including immunodominant membrane protein (Imp), immunodominant membrane protein A (IdpA), and antigenic membrane protein (Amp) (Kakizawa et al., 2006b). These proteins are highly variable in the amino acid sequences due to the selective pressure from interactions with the environment and hosts (Kakizawa et al., 2006a, 2009). Previous studies show that the Imp of ‘*Ca.* P. mali’ can interact with the actin of plant hosts (Boonrod et al., 2012); the Amp of ‘*Ca.* P. asteris’ can interact with the actin of insect vectors and form the Amp-microfilament complex together with myosin (Suzuki et al., 2006; Galetto et al., 2011). These interactions seem to play important roles in determining the mobility of phytoplasma within plant hosts and the transmissibility of phytoplasma by insect vectors (Konnerth et al., 2016).

Despite having small genomes compared with other bacteria, phytoplasmas contain high numbers of repetitive genes lying within potential mobile unit (PMU) or sequence-variable mosaic (SVM) regions, which are proposed to be remnants of transposons or prophage (Bai et al., 2006; Wei et al., 2008). These regions are flanked by inverted repeats and contain genes involved in DNA recombination (*tra5*, *ssb*, *himA*) and replication (*dnaG*, *dnaB*), suggesting that they have the ability to transpose within the genome and be horizontally transferred among phytoplasmas (Toruno et al., 2010; Chung et al., 2013; Ku et al., 2013a; Cho et al., 2019). The repeat-rich nature of phytoplasma genomes is proposed to contribute to the frequent recombination events, leading to the considerable variation in genome sizes among phytoplasmas (Kube et al., 2008; Andersen et al., 2013; Marcone, 2014; Seruga Music et al., 2019). Moreover, as putative pathogenicity islands, these regions often carry effector genes and are proposed to contribute to the adaptation of phytoplasma switching in plant and insect hosts (MacLean et al., 2011; Oshima et al., 2011; Chung et al., 2013).

The availability of complete genomes not only provides a better chance to identify potential genes involved in phytoplasma–host interactions, but it also contributes to a better understanding of the phytoplasma genome organization and evolution (Hogenhout and Seruga Music, 2009; Kube et al., 2012; Marcone, 2014). In the past, phytoplasma DNA was enriched via procedures, such as cesium chloride density gradient centrifugation and pulse-field gel electrophoresis (PFGE) (Hogenhout and Seruga Music, 2009). However, the density gradient centrifugation method may also enrich plant chloroplast DNA and reduce DNA integrity, and the PFGE method typically has low yields. Given the difficulties in isolating phytoplasma DNA from an infected host, together with the fact that phytoplasmas have AT- and repeat-rich genomes, only seven complete genome sequences of phytoplasmas were released over 15 years since the first Onion Yellows phytoplasma strain M (OY-M) was completed in 2004 (Oshima et al., 2004; Bai et al., 2006; Kube et al., 2008; Tran-Nguyen et al., 2008; Andersen et al., 2013; Orlovskis et al., 2017; Wang J. et al., 2018). These genome sequences represent only four 16S rRNA gene RFLP (16Sr) groups, including 16SrI, 16SrX, 16SrXII, and 16SrV.

Recently, with the introduction of Illumina sequencing technology and decreasing cost, more than 30 phytoplasma draft genome sequences were published. Those draft genomes were determined without further enrichment of phytoplasma DNA and utilized selective exclusion of the host reads by using the healthy plant genome as a reference or selective inclusion of phytoplasma reads using available phytoplasma genome sequences as references (Chung et al., 2013; Polano and Firrao, 2018). Although draft genome sequences provide insights into phytoplasma biology, the fragmented nature of draft genomes limits the type of comparative genomics analysis that can be conducted (Ricker et al., 2012). Previously, a draft genome of ‘*Ca.* P. aurantifolia’ NCHU2014 (16SrII group) associated with *Echinacea purpurea* witches’ broom (EpWB) disease was obtained based on Illumina paired-end sequencing, which contains 28 contigs with a combined size of 545,427 bp and encodes 433 protein-coding genes (Chang et al., 2015). In this study, an integrated solution that combined immunoprecipitation-based enrichment of phytoplasma cells prior to DNA extraction and Oxford Nanopore Technologies (ONT) MinION long-read DNA sequencing was developed to obtain a complete genome sequence of ‘*Ca*. P. aurantifolia’ NCHU2014. Furthermore, with the complete genome sequence of this strain determined, we conducted comparative analysis with other phytoplasmas and experimental characterization of its effectors.

## Materials and Methods

### Polyclonal Antibody Production

A codon-optimized DNA fragment encoding the Imp of ‘*Ca*. P. aurantifolia’ NCHU2014 without the transmembrane domain was subcloned into the SUMO-pET vector and introduced into *Escherichia coli* BL21 (DE3). The N-terminal His-SUMO tagged ImpΔN protein was produced at 24°C by isopropyl β-D-1-thiogalactopyranoside induction and purified by Ni^2+^-NTA resin (Qiagen) according to the manufacturer’s instructions. The purified protein was cleaved with Ubiquitin-like-specific protease 1 and then reapplied to Ni^2+^-NTA resin for removing the cleaved His-SUMO tag and uncleaved His-SUMO-ImpΔN. The recombinant ImpΔN was obtained in the flowthrough and prepared for polyclonal antibody production in rabbits.

### Affinity Purification of ‘*Ca*. P. aurantifolia’ NCHU2014

The antibody-based purification was developed to enrich ‘*Ca*. P. aurantifolia’ NCHU2014, which was maintained in periwinkle (*Catharanthus roseus*) by grafting. The stems and leaf veins of symptomatic periwinkle were sampled and gently homogenized with liquid nitrogen using mortar and pestle. Subsequently, 3 g of grinded tissues were suspended with 6 ml PBS buffer (0.137 M NaCl, 2.7 mM KCl, 10 mM Na_2_HPO_4_, 1.8 mM KH_2_PO_4_, pH 6.8) and filtrated with a cell strainer (100 μm) to remove unfragmented tissues. Meanwhile, the anti-Imp antibody-coated beads were prepared by incubating antiserum (containing polyclonal anti-Imp antibodies) and Novex^®^ Dynabeads Protein A (superparamagnetic beads with recombinant Protein A covalently coupled to the surface). The flowthrough containing homogenized cells was incubated with antibody-coated beads (3 mg) for 30 min at 4°C, and then the unwanted substances were separated from the beads by magnet. The remaining materials containing phytoplasma cells were washed extensively with PBS buffer and collected for genomic DNA extraction.

### MinION-Based DNA Sequencing

After the affinity-purified cells enriched from 3 g of grinded tissues were lysed, about 6 μg genomic DNA was obtained from extraction by the Plant Genomic DNA Purification Kit (Gene Mark, Taiwan) according to the manufacturer’s instructions. After further cleaning by the Quick-DNA Plant/Seed Miniprep Kit (ZYMO RESEARCH), approximately 4 μg of DNA was obtained from one sample with 3 g of starting plant materials. A total of 2 μg genomic DNA was used for each library construction by the KAPA Hyper Prep Kit optimization for Nanopore 1D (Oxford Nanopore Technologies, Oxford, United Kingdom) according to the manufacturer’s instructions. To perform long-read sequencing, DNA libraries were loaded on MinION flow cells (R9.4), and the Nanopore reads were base-called from FAST5 files using the ONT Albacore Software (version 2.0.1).

### Genome Assembly and Annotation

The procedures for genome assembly and annotation were based on those described in our previous studies (Chung et al., 2013; Cho et al., 2020). All bioinformatics tools were used with the default settings unless stated otherwise. Briefly, the MinION reads were mapped to a previously published draft genome (Chang et al., 2015) using Minimap2 v2.15 (Li, 2018). Visual inspection of the mapping result using IGV v2.3.57 (Robinson et al., 2011) produced one circular scaffold representing the chromosome and one circular contig representing the plasmid. Next, an iterative process was used to complete and validate the assembly. In each iteration, the MinION reads were mapped as described, and the Illumina reads from our previous study (Chang et al., 2015) were mapped to the assembly using BWA v0.7.12 (Li and Durbin, 2009). The raw read mapping results were programmatically checked using SAMtools v1.2 (Li et al., 2009) and manually inspected using IGV v2.3.57 (Robinson et al., 2011). The iterative process was repeated until all polymorphic sites were resolved and a complete assembly was obtained. For the assembly process, only the MinION reads from runs A01 to A10 (Table 1) were used. The MinION reads from runs B01 to B03 were only used for checking coverage after the complete assembly was validated.

**TABLE 1:** Raw reads statistics.

**Run**	**Read count**	**Sequencing output (bp)**	**Max. length (bp)**	**Av. length (bp)**	**Reads mapped to phytoplasma genome**	**% Reads mapped to phytoplasma genome**	**Reads mapped to chloroplast genome**	**% Reads mapped to chloroplast genome**	**Reads mapped to plant nuclear genome**	**% Reads mapped to plant nuclear genome**	**% Unmapped reads**	**Phytoplasma genome coverage (bp)**	**Phytoplasma genome sequencing depth (fold)**
A01	148,631	554,291,700	46,543	3,729	53,487	36.0	11,474	7.7	78,134	52.6	3.7	639,808	209
A02	88,111	347,096,923	41,777	3,939	30,963	35.1	6,565	7.5	47,564	54.0	3.4	639,808	126
A03	58,938	243,448,556	40,264	4,131	20,511	34.8	4,179	7.1	32,044	54.4	3.7	639,808	87
A04	14,718	61,459,870	46,555	4,176	5,165	35.1	1,092	7.4	7,834	53.2	4.3	639,808	22
A05	23,978	95,643,639	34,810	3,989	8,495	35.4	1,678	7.0	12,802	53.4	4.2	639,808	34
A06	533,668	2,653,344,966	55,606	4,972	119,855	22.5	44,280	8.3	353,160	66.2	3.1	639,808	605
A07	292,158	1,537,572,472	52,917	5,263	74,261	25.4	24,101	8.2	185,853	63.6	2.7	639,808	384
A08	161,139	771,282,841	39,480	4,786	48,419	30.0	13,476	8.4	94,699	58.8	2.8	639,808	244
A09	36,062	156,720,727	41,438	4,346	11,348	31.5	3,358	9.3	20,183	56.0	3.3	639,808	51
A10	28,469	119,536,595	44,746	4,199	9,168	32.2	2,721	9.6	15,516	54.5	3.7	639,808	41
Subtotal	1,385,872	6,540,398,289			381,672	27.5	112,924	8.1	847,789	61.2	3.1		1,802
B01	207,087	509,103,092	61,271	2,458	34,235	16.5	8,341	4.0	142,745	68.9	10.5	602,845	47
B02	25,499	127,421,361	44,713	4,997	2,043	8.0	1,001	3.9	19,672	77.1	10.9	613,315	8
B03	475,713	2,194,510,560	91,125	4,613	110,450	23.2	16,647	3.5	314,627	66.1	7.1	619,020	194
Subtotal	708,299	2,831,035,013			146,728	20.7	25,989	3.7	477,044	67.4	8.3		249
C01	34,321,466	10,206,843,846	300	297	5,477,697	16.0	2,265,456	6.6	26,462,971	77.1	0.3	637,986	2,117

*Runs A01–A10 were based on the ONT sequencing with the immunoprecipitation enrichment of phytoplasma cells; four biological samples for runs A01–A02, A03–A05, A06–07, and A08–A10, respectively. Runs B01–B03 were based on the ONT sequencing without the enrichment; each used a different biological sample. Run C01 was based on the Illumina paired-end sequencing from our previous study (DOI: 10.1128/genomeA.01398-1); no enrichment was performed.*

For annotation, RNAmmer v1.2 (Lagesen et al., 2007), tRNAscan-SE v1.3.1 (Lowe and Eddy, 1997), and Prodigal v2.6.3 (Hyatt et al., 2010) were used for gene prediction. The homologous genes in other representative phytoplasma genomes (Table 2) were identified using OrthoMCL (Li et al., 2003) to provide gene name and product description. Gene encoding putative secreted proteins were identified based on two established procedures, including one that uses SignalP v4.1 (Petersen et al., 2011) as described in Garcion et al. (2021) and another that uses SignalP v5.0 (Armenteros et al., 2019) as described in Cho et al. (2019). Additionally, BlastKOALA (Kanehisa et al., 2016) and GenBank (Benson et al., 2018) sequence similarity searches were used for manual curation of the annotation results.

**TABLE 2:** List of the representative phytoplasma genome sequences analyzed.

**Strain**	**16Sr Group**	**Accession**	**Chromosome**	**Size (bp)**	**G + C Content (%)**	**Coding density (%)**	**Coding sequences**	**Pseudogenes**	**tRNA genes**	**rRNA genes**
‘*Ca.* P. aurantifolia’ NCHU2014	II	CP040925	Circular	635,584	24.5	66.3	471	35	24	6
‘*Ca.* P. luffae’ NCHU2019	VIII	CP054393	Circular	769,143	23.3	80.3	725	13	31	6
‘*Ca.* P. ziziphi’ Jwb-nky	V	CP025121	Circular	750,803	23.2	75.4	640	31	32	6
‘*Ca.* P. mali’ AT	X	CU469464	Linear	601,943	21.4	76.7	482	15	32	6
‘*Ca.* P. australiense’ PAa	XII	AM422018	Circular	879,959	27.4	64.1	684	155	35	6
‘*Ca.* P. asteris’ AYWB	I-A	CP000061	Circular	706,569	26.9	73.5	671	0	31	6
‘*Ca.* P. asteris’ OY-M	I-B	AP006628	Circular	853,092	27.8	73.0	752	0	32	6

*The pseudogene counts are based on those annotated in the standard format (i.e., with the “/pseudo” tag). For ‘*Ca*. P. asteris’ OY-M, 46 coding sequences are annotated as “possible pseudogene” in the “/note” field.*

For quality check, the raw reads that were not mapped to the phytoplasma genome were collected and mapped to the host (*Catharanthus roseus*) genome in two steps using Minimap2 v2.15 (Li, 2018). The first step mapped to the chloroplast genome (accession KC561139) (Ku et al., 2013b), and the second step mapped to the host nuclear genome (accession JQHZ01000000) (Kellner et al., 2015).

### Co-expression Assays

A codon-optimized DNA fragment encoding SAP11 of ‘*Ca*. P. aurantifolia’ NCHU2014 without the signal peptide was subcloned into a binary vector pBA002 for expression under the control of the *CaMV* 35S promoter. Plasmids for expression of the N-terminal FLAG-tagged *Arabidopsis* TCP transcription factors (SFP-AtT) were obtained as previously described (Chang et al., 2018). *Nicotiana benthamiana* grown at 26°C was used for transient co-expression assays. A mixture of *A. tumefaciens* strain ABI carrying the desired constructs of *35S:SAP11* and *35S:SFP-AtTCPs* was introduced into *N. benthamiana* leaves by agroinfiltration. After 2 days, samples prepared from two infiltrated leaves (the third and fourth leaves counting from the top of 4- to 5-week-old plants) were collected for Western blotting analysis.

### Western Blotting

Collected samples were ground into powder using liquid nitrogen. About 0.1 g sample powder was added to 0.2 ml 2.5X SDS sample buffer (5 mM EDTA, 5% SDS, 0.3 M Tris-HCl, pH 6.8, 20% glycerol, 1% β-mercaptoethanol, and bromophenyl blue) and heated in a boiling water bath for 5 min. After centrifugation, supernatants were obtained as total cell extracts, and proteins were separated by SDS-PAGE. Antibodies against Imp (polyclonal), SAP11 (polyclonal), and FLAG^TM^ tag (monoclonal) were used to monitor protein amounts. Chemiluminescence signals generated by Amersham ECL reagents were captured using the ImageQuant LAS 4000 mini (GE Healthcare).

## Results

### Immunoprecipitation-Based Enrichment of ‘*Ca*. P. aurantifolia’ NCHU2014

**FIGURE 1:**
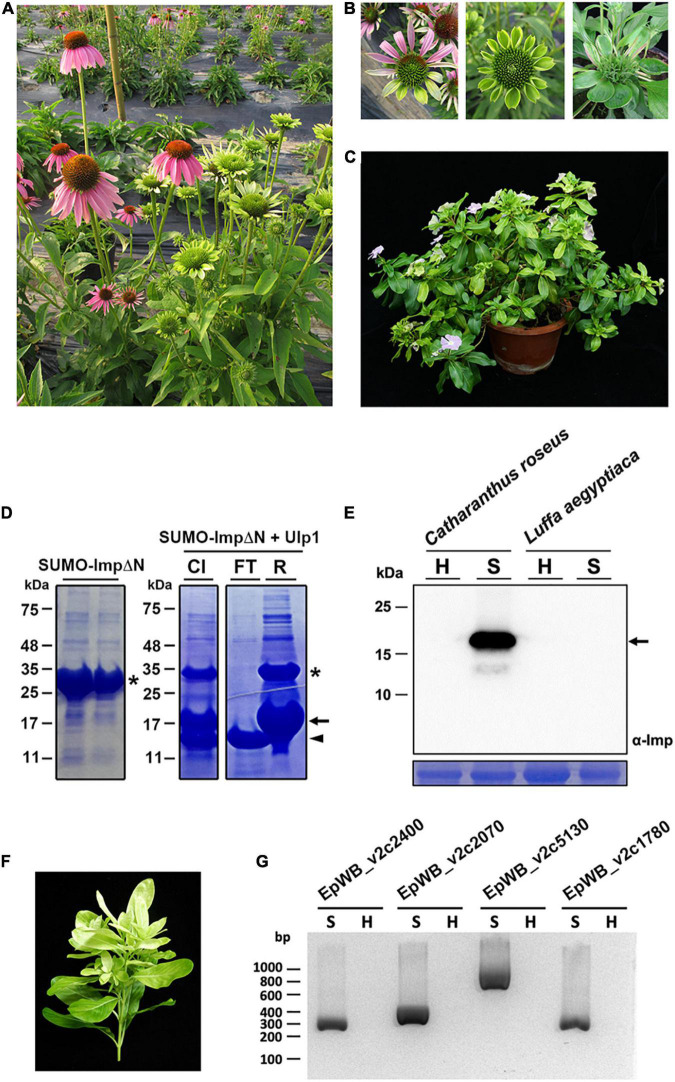
Immunoprecipitation-based enrichment of ‘*Ca*. P. aurantifolia’ NCHU2014. **(A)** Phenotypic comparisons between healthy (left) and ‘*Ca*. P. aurantifolia’ NCHU2014-infected (right) purple coneflower (*Echinacea purpurea*). The diseased plants exhibited phyllody and witches’ broom symptoms. **(B)** Enlarged images of the abnormal flowers carrying phyllody, virescence, and bud proliferation symptoms in ‘*Ca*. P. aurantifolia’ NCHU2014-infected purple coneflower. **(C)** Symptomatic periwinkle (*Catharanthus roseus*) associated with ‘*Ca*. P. aurantifolia’ NCHU2014 transmitted by dodder. **(D)** The His-SUMO tagged ImpΔN was expressed in *E. coli* and purified by Ni^2+^-NTA resin (left panel). After cleavage of the His-SUMO tag by Ulp1 (CI), the reaction mixture was applied on the Ni^2+^-NTA column. Arrowhead indicates the purified ImpΔN in flowthrough (FT); asterisk and arrow indicate the uncleaved His-SUMO-ImpΔN and His-SUMO, respectively, in the resin (R). **(E)** Total cell extracts prepared from healthy (H) and symptomatic (S) leaves were examined by Western blotting using specific antibody against Imp of ‘*Ca*. P. aurantifolia’ NCHU2014. The specific signal of Imp was only detected in symptomatic *C. roseus* infected with ‘*Ca*. P. aurantifolia’ NCHU2014, but not symptomatic loofah (*Luffa aegyptiaca*) infected with ‘*Ca*. P. luffae’ NCHU2019 (upper panel). As a loading control, the large subunit of Rubisco was visualized with Coomassie Brilliant Blue staining (lower panel). Arrow indicates the 19 kDa Imp. **(F)** The ‘*Ca*. P. aurantifolia’ NCHU2014-infected periwinkle used for immunoprecipitation. **(G)** PCR examination of DNA extracted from the immunoprecipitated fraction containing ‘*Ca*. P. aurantifolia’ NCHU2014.

The ‘*Ca*. P. aurantifolia’ NCHU2014 was originally collected from purple coneflower (*Echinacea purpurea*) in Taiwan and transferred to periwinkle by dodder (Chang et al., 2015). The diseased plants infected with ‘*Ca*. P. aurantifolia’ NCHU2014 exhibited phyllody, virescence, and witches’ broom phenotypes in which the development of leaf-like structures with the loss of flower pigment and the proliferation of shoots in place of carpels were observed (Figures 1A–C).

As an obligate intracellular parasite, it is challenging to obtain a complete genome sequence of phytoplasma due to the host DNA contamination. To enrich ‘*Ca*. P. aurantifolia’ NCHU2014 for whole genome sequencing, immunoprecipitation-based purification of phytoplasma cells was conducted using the polyclonal antibody raised against the Imp of ‘*Ca*. P. aurantifolia’ NCHU2014. The antibody was generated in rabbit using the recombinant protein of Imp without N-terminal transmembrane domain (Figure 1D), which specifically recognized the 16SrII group ‘*Ca*. P. aurantifolia’ NCHU2014 in symptomatic periwinkle but not the 16SrVIII group ‘*Ca*. P. luffae’ NCHU2019 associated with loofah witches’ broom disease (Figure 1E and Supplementary Figure 1). For affinity purification of ‘*Ca*. P. aurantifolia’ NCHU2014, the stems and leaf veins from the symptomatic periwinkle displaying chlorosis, witches’ broom, phyllody, and virescence were used (Figure 1F). Genomic DNA extracted from the immunoprecipitated fraction containing ‘*Ca*. P. aurantifolia’ NCHU2014 was obtained for PCR examination. The PCR products specific for EpWB_v2c2400, EpWB_v2c2070, EpWB_v2c5130, and EpWB_v2c1780 genes of ‘*Ca*. P. aurantifolia’ NCHU2014 encoding SAP54/phyllogen, putative secreted protein, Mn/Zn-binding protein, and 50S ribosomal protein L17, respectively, were amplified only with the DNA sample extracted from symptomatic plants but not healthy plants (Figure 1G). The extracted DNA was then further processed for high-throughput DNA sequencing.

### MinION-Based Long-Read DNA Sequencing and Genome Assembly

**FIGURE 2:**
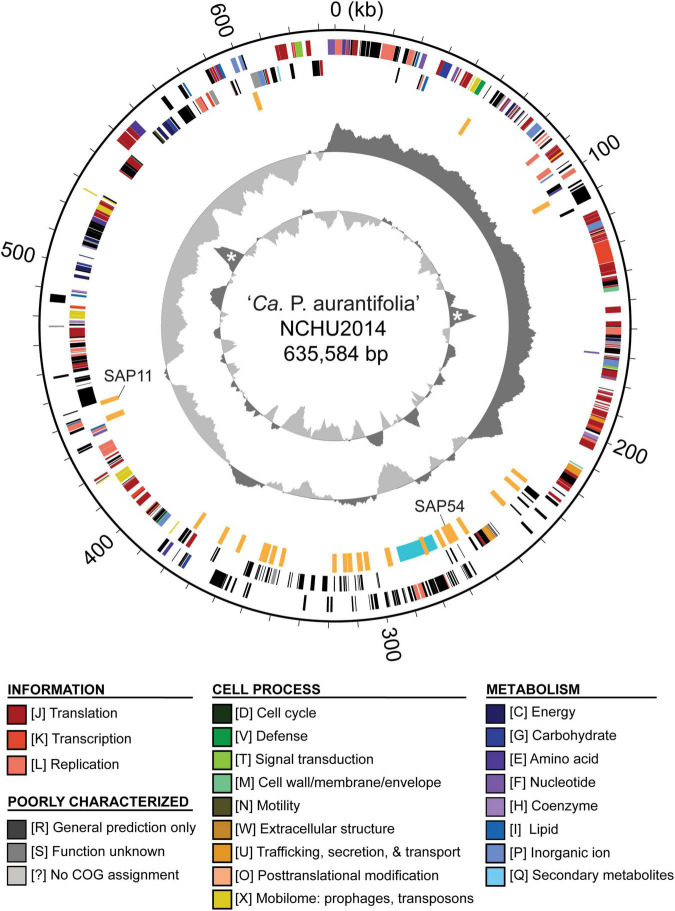
Genome map of ‘*Ca*. P. aurantifolia’ NCHU2014. Rings from outside in: (1) scale marks; (2 and 3) coding sequences on the forward and reverse strand, respectively (color-coded by functional categories); (4) putative effectors (orange) and putative mobile unit (light blue); (5) GC skew (positive: dark gray; negative: light gray); (6) GC content (above average: dark gray; below average: light gray), two peaks corresponding to the rRNA operons are marked by “*”.

To improve the draft genome assembly of ‘*Ca*. P. aurantifolia’ NCHU2014 (Chang et al., 2015), we used ONT MinION to generate long sequencing reads. A total of 1,385,872 reads containing 6,540,398,289 bp were obtained from the libraries constructed using four DNA samples from our enrichment procedure (Table 1). These reads were obtained from 10 sequencing runs using two MinION flow cells. The first flow cell was used for two libraries (runs A01–A02 and A03–A05, respectively), producing 334,376 reads totaling 1,301,940,688 bp. The second flow cell was used for two additional libraries (runs A06–A07 and A08–A10, respectively), producing 1,051,496 reads totaling 5,238,457,601 bp. The average read lengths ranged from 3.7 to 5.2 kb with a maximum read length of 55.6 kb. Using these newly generated long reads, combined with our previous Illumina data set (Chang et al., 2015), we obtained a complete genome assembly for ‘*Ca*. P. aurantifolia’ NCHU2014. This assembly contains one circular chromosome with 635,584 bp and one circular plasmid with 4,224 bp (Figure 2). To the best of our knowledge, this is the first complete genome available for 16SrII phytoplasma.

Compared with the previous Illumina-only draft assembly of the same strain (Chang et al., 2015), 23 chromosomal regions ranging from 214 to 32,404 bp in size are newly assembled; the plasmid is unchanged. One 193-bp region (positions 354,755–354,947 of the chromosome) is shown as a gap based on the ONT reads but is supported by the Illumina reads. One possible explanation is that this region was deleted between our sample collections for Illumina (in 2014) and ONT sequencing (in 2019). Additionally, by examining the mapping results of Illumina reads to this complete assembly, we found that 15 chromosomal regions (mostly 36–128 bp in size, totaling 1,822 bp) are imperfect repeats and have no Illumina reads mapped.

As a quality evaluation, we also performed MinION sequencing to generate 708,299 reads (B01–B03; three runs totaling 2,831,035,013 bp using one MinION flowcell) using the libraries constructed with three DNA samples obtained without the immunoprecipitation-based enrichment. Compared with the enrichment protocol from which 22.5 to 36.0% of the raw reads were from phytoplasma, only 8.0 to 23.2% of the raw reads were from phytoplasma without the enrichment (Table 1). Importantly, the reads obtained with the enrichment procedure can cover all base pairs of the assembled phytoplasma genome even when only 14,718 reads were obtained in run A04 (Table 1). In contrast, up to approximately 37 kb of the phytoplasma genome were not covered by reads obtain from unenriched samples (B01). Based on these results, our enrichment procedure provides substantial improvements applicable to phytoplasma genome research.

### Genome Comparison Between Two Closely Related ‘*Ca*. P. aurantifolia’ Strains

**FIGURE 3:**
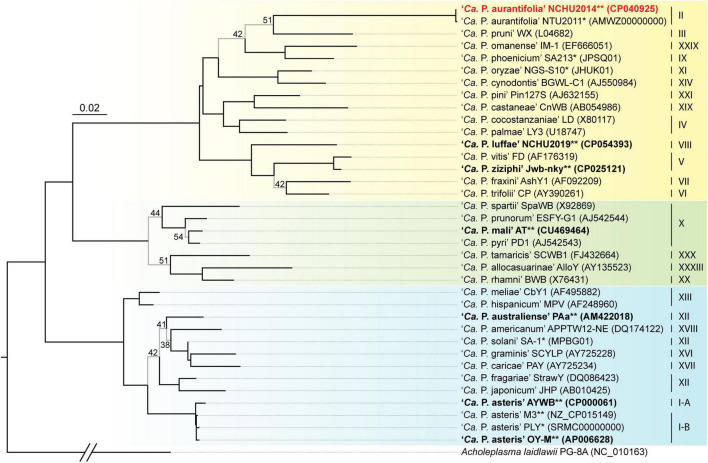
Maximum likelihood phylogeny of representative phytoplasmas based on the 16S rRNA genes. The internal branches with < 60% bootstrap support are drawn in light gray, and the support values are labeled. Strains with genome sequences available are highlighted (“*”, draft; “**”, complete), those included in the genome comparisons are in bold. The focal strain of this study, ‘*Ca*. P. aurantifolia’ NCHU2014, is highlighted in red. Sequence accession numbers are provided in parentheses following the strain names. For genome sequences with two 16S rRNA gene sequences available, the first one (i.e., the one with a smaller numerical value in locus tag) was selected. The 16Sr group assignments are provided on the right. Background colors are used to highlight the three phylogenetic clusters of phytoplasmas (I: blue; II; green; III: yellow). *Acholeplasma laidlawii* is included as the outgroup.

**FIGURE 4:**
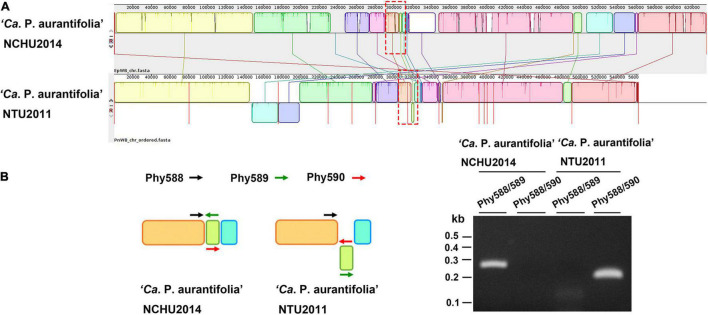
Pairwise alignment of linearized genomes of ‘*Ca*. P. aurantifolia’ strains NCHU2014 and NTU2011. **(A)** The complete genome of ‘*Ca*. P. aurantifolia’ NCHU2014 was aligned with the draft genome of ‘*Ca*. P. aurantifolia’ NTU2011. **(B)** Schematic diagram represented the inversion rearrangements indicated by red-dashed rectangles in **(A)** and the specific primers designed for PCR assays (left). The DNA fragments corresponding to the breakpoint of inverted structures in both chromosomal genomes were amplified (right). Primers: Phy588 (5′-GTTACTTTCGTGATTAATTCC-3′), Phy589 (5′-GAATTTACAACAAGCTCAATTGG-3′), Phy560 (5′-CTTGACTAATTTACTTTCGTCGC-3′).

A phylogenetic tree based on 16S ribosomal RNA (16S rRNA) genes of representative phytoplasmas was inferred to illustrate the evolutionary relationships (Figure 3). As the first complete genome sequence available for 16SrII group phytoplasma, ‘*Ca*. P. aurantifolia’ NCHU2014 shares 100% sequence identity of the 16S rRNA gene with ‘*Ca*. P. aurantifolia’ NTU2011. ‘*Ca*. P. aurantifolia’ NTU2011 was originally collected from infected peanut plants associated with peanut witches’ broom (PnWB) disease in Taiwan. So far, only a draft genome was available for ‘*Ca*. P. aurantifolia’ NTU2011, which contains 13 chromosomal contigs with a combined size of 562,473 bp and a plasmid with 4,221 bp (Chung et al., 2013). Comparative genomic analysis between two closely related strains revealed that the average nucleotide identity (ANI) is 99.72% across 90.79% chromosomal segments. This result reflects a high genomic similarity between these two strains in Taiwan. However, the draft genome of NTU2011 lacks two large segments corresponding to positions 232,370–246,973 and 313,259–348,010 of the NCHU2014 chromosome (Supplementary Figure 2). Further analysis by the pairwise genome alignment revealed that the chromosomes of these two strains are largely collinear although some rearrangements, including inversions and translocations, were observed (Figure 4A). The rearranged regions were confirmed experimentally by PCR analysis (Figure 4B).

The plasmids found in these two strains are nearly identical with 99.13% sequence identity. Only four protein-coding genes were found, including those corresponding to one replication protein, one DNA primase, one threonine synthase, and one hypothetical protein. Our BLASTN search against the NCBI Nucleotide Collection (nt) database did not find any similar sequence available for comparative analysis.

### Comparative Analysis of Gene Content of ‘*Ca*. P. aurantifolia’ NCHU2014

**FIGURE 5:**
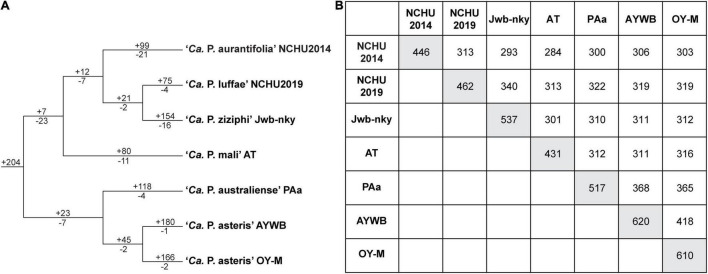
Distribution patterns of homologous gene clusters. **(A)** Phylogenetic distribution. The cladogram was based on a maximum likelihood phylogeny inferred using 204 shared single-copy genes; the concatenated alignment contains 72,653 aligned amino acid sites. Numbers above a branch and preceded by a “ + ” sign indicate the number of clusters that are uniquely present in all daughter lineages; numbers below a branch and preceded by a “–” sign indicate the number of clusters that are uniquely absent. **(B)** Numbers on the diagonal indicate the counts of homologous gene clusters found in each of the *Candidatus* species genomes; numbers above the diagonal indicate the counts of homologous gene clusters shared in pairwise comparisons.

The ‘*Ca*. P. aurantifolia’ NCHU2014 genome consists of one circular chromosome with 635,584 bp and one plasmid with 4,224 bp. Based on the annotation, the chromosome encodes six rRNA genes, 24 tRNA genes, 471 protein-coding genes, and 35 pseudogenes (Table 2). Notably, ‘*Ca*. P. aurantifolia’ NCHU2014 has a low coding density (66.3%) with the lowest numbers of tRNA and protein-coding genes compared with other phytoplasmas with complete genome sequences available (Table 2). Among the protein-coding genes, only 352 (75%) genes were assigned to COG categories with specific functions (Figure 2). There were 119 (25%) genes annotated as hypothetical proteins without COG functional category assignments. Similar to other phytoplasmas with complete genome information, ‘*Ca*. P. aurantifolia’ NCHU2014 has a small chromosome with low GC content (24.5%) and lacks the genes for many metabolic pathways although it has 33 genes annotated as transporters. The comparative analysis further revealed that ‘*Ca*. P. aurantifolia’ NCHU2014 share 284–313 homologous gene clusters with other lineages surveyed (Figure 5A). Among them, a conserved core of 204 homologous gene clusters was found in all lineages surveyed (Figure 5B). This is consistent with other obligate parasitic bacteria that pose a high level of genomic plasticity (Andersen et al., 2013).

### Effectors (Virulence-Related Factors) and Potential Mobile Units

Phytoplasmas possess the Sec secretion system for transportation of effectors into the host cell cytoplasm (Sugio et al., 2011b; Oshima et al., 2013). Based on the prediction of N-terminal signal peptide by SignalP-5.0, 28 putative secreted proteins were identified (Supplementary Table 1). Surprisingly, when SignalP-4.1 was used, one of these 28 (EPWB_v2c3230) was excluded, and 70 additional putative secreted proteins were identified. Our manual inspection of these prediction results found that many of the putative secreted proteins identified by SignalP-4.1 are likely to be false positives (e.g., ribosomal proteins). In comparison, only one of the 28 candidates identified by SignalP-5.0 was an obvious false positive (EPWB_v2c2520; ATP-dependent Zn protease). Based on these findings, 27 putative secreted proteins predicted by SignalP-5.0 (excluding EPWB_v2c2520) were used for downstream analysis.

**FIGURE 6:**
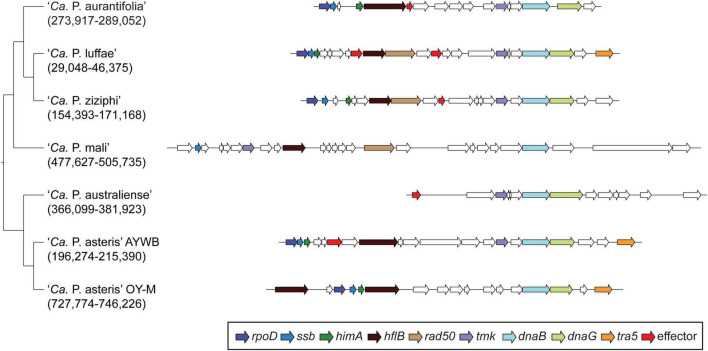
Gene organization of PMUs. One representative PMU was selected from each genome for visualization, the chromosomal locations are labeled below the *Candidatus* species names. PMU-associated genes are color-coded according to annotation. Additionally, putative effector genes are colored in red. Intact genes are drawn with solid borders, putative pseudogenes are drawn with dotted borders.

**FIGURE 7:**
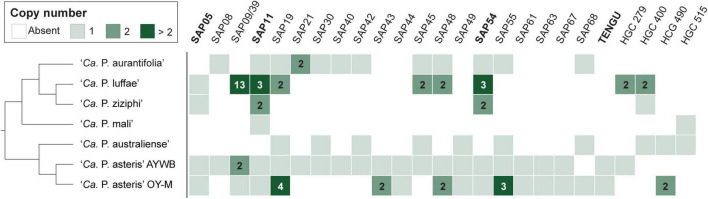
Distribution of genes that encode putative secreted proteins among representative phytoplasmas. Only those found in at least two genomes are presented, the gene copy numbers are presented in the form of a heat map. The gene names are labeled according to the ‘*Ca*. P. asteris’ isolates AYWB or OY-M annotation when available; the remaining ones are labeled according the homologous gene cluster (HGC) IDs of this work. Those effectors genes that have been experimentally characterized (i.e., SAP05, SAP11, SAP54, and TENGU) are highlighted in bold. ‘*Ca*. P. aurantifolia’ NCHU2014 has two divergent SAP05 homologs (locus tags EPWB_v2c2740 and EPWB_v2c3230) that do not meet the criteria for inclusion in the figure.

Among these 27 candidates, only one (EpWB_v2c2530) with uncharacterized function was found in PMU (Figure 6), which is different from the previous report that the many of the 56 secreted AYWB protein (SAP) genes in ‘*Ca*. P. asteris’ were associated with PMUs (Bai et al., 2009). This result may be explained by the fact that only one PMU was found in ‘*Ca*. P. aurantifolia’ NCHU2014, which contains PMU-associated genes (*dnaG*, *dnaB*, *tmk*, *smc*, *hflB*, *himA, ssb, and rpoD*) and is closely related to those of ‘*Ca*. P. luffae’ NCHU2019 and ‘*Ca.* P. ziziphi’ Jwb-nky in gene content and organization (Figure 6). In comparison, the chromosome of ‘*Ca*. P. asteris’ AY-WB harbors at least five PMU regions (Bai et al., 2006, 2009). Nevertheless, two thirds of putative secreted protein genes found in ‘*Ca*. P. aurantifolia’ NCHU2014 formed clusters and closely located on both sides of PMU (Figure 2). In addition, 12 out of the 13 SAP homologs in the NCHU2014 genome are located in these clusters. The only exception is the homolog of SAP11 (SAP11_E__pWB_; EPWB_v2c3970), which is located far from these clusters. Comparison of putative effector gene content revealed that ‘*Ca*. P. aurantifolia’ NCHU2014 harbors SAP11 (Bai et al., 2009) and SAP54 (MacLean et al., 2011) homologs while lacking homologs o SAP05 (Huang et al., 2021) or TENGU (Hoshi et al., 2009; Figure 7 and Supplementary Table 2).

### Phytoplasma SAP11_E__pWB_ Destabilizes *Arabidopsis* Class II CYC/TB1-TCPs

**FIGURE 8:**
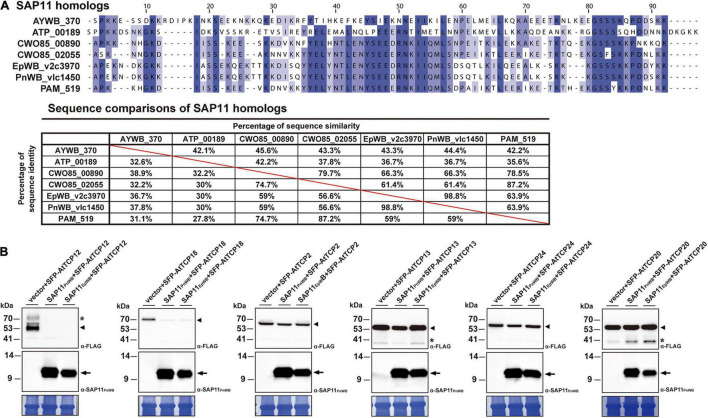
Destabilization of *Arabidopsis* CYC/TB1-TCPs transcription factors by SAP11 of ‘*Ca*. P. aurantifolia’ NCHU2014. **(A)** Sequence comparison of SAP11 homologs identified in ‘*Ca*. P. asteris’ AYWB (AYWB_370), ‘*Ca*. P. mali’ AT (ATP_00189), ‘*Ca*. P. ziziphi’ Jwb-nky (CWO85_00890), ‘*Ca*. P. ziziphi’ Jwb-nky (CWO85_02055), ‘*Ca*. P. aurantifoli’ NCHU2014 (EpWB_v2c3970), ‘*Ca*. P. aurantifolia’ NTU2011 (PnWB_v1c1450), and ‘*Ca*. P. asteris’ OY-M (PAM_519). SAP11 homologs without the signal peptide were aligned by MEGA 7.0 using ClustalW. The sequence alignment was then edited by Jalview software in which identical residues are shaded in blue (upper panel). The color gradient indicates the level of sequence conservation at each position. The sequence identity and sequence similarity between SAP11 homologs are presented on the lower panel. **(B)** After transient co-expression in *N. benthamiana*, the relative abundance levels of FLAG-tagged PCF-TCP (AtTCP20), CIN-TCPs (AtTCP2, AtTCP13, and AtTCP24), and CYC/TB1-TCPs (AtTCP12 and AtTCP18) were examined in the presence of SAP11 effectors through Western blotting. Monoclonal antibody against the FLAG-tagged TCPs (upper panel) and polyclonal antibody against SAP11 effectors (middle panel) were used. As a loading control, the large subunit of Rubisco was visualized with Coomassie Brilliant Blue staining (lower panel). Non-specific bandings recognized by antibodies are indicated by asterisks.

Previously, it has been demonstrated that SAP11 has the ability to destabilize CYC/TB1 (CYCLOIDEA/TEOSINTE-BRANCHED1)-TCPs (TCP12 and TCP18), leading to the proliferation of axillary meristems (Chang et al., 2018; Wang N. et al., 2018; Pecher et al., 2019). The SAP11_E__pWB_ identified in ‘*Ca*. P. aurantifolia’ NCHU2014 shares a high degree of amino acid sequence identity (98.8%) with SAP11_P__nWB_ but only has 36.7% sequence identity with SAP11_A__YWB_ (Figure 8A). To understand the ability of SAP11_E__pWB_ in destabilizing TCP transcription factors, co-expression of SAP11_E__pWB_ and FLAG-tagged *A. thaliana* TCPs were conducted in *N. benthamiana* using agroinfiltration. Similar to SAP11_P__nWB_, SAP11_E__pWB_ exhibited a strong ability to destabilize class II CYC/TB1-TCPs (AtTCP12 and AtTCP18) but did not destabilize class II CIN-TCPs (AtTCP2, AtTCP13, and AtTCP24) as well as class I PCF-TCP (AtTCP20) (Figure 8B). As a control, the AtTCP12 and AtTCP18 were not decreased in abundance with the presence of the vector alone. Despite the fact that SAP11_E__pWB_ display one amino acid difference with SAP11_P__nWB_ in the C-terminal end, the potential biochemical activities in destabilizing CYC/TB1-TCP transcription factors were similar to each other. This is consistent with the fact that SAP11 lacking C-terminus (SAP11ΔC) still has the abilities for TCP binding and destabilization (Sugio et al., 2014). Because another phytoplasma effector, TENGU, with the ability to enhance the proliferation of axillary meristems, was not found in ‘*Ca*. P. aurantifolia’ NCHU2014, SAP11_E__pWB_ would be the core virulence factor to induce witches’ broom symptom in diseased plants (Figures 1A–C).

## Discussion

In general, phytoplasmas contain one circular chromosome smaller than 1,000 kb in size (Oshima et al., 2013). In this study, we found that ‘*Ca*. P. aurantifolia’ NCHU2014 has the smallest circular chromosome with 635,584 bp among the phytoplasmas with complete genome sequences available, and ‘*Ca*. P. mali’ AT has an even smaller linear chromosome with 601,943 bp (Table 2). In the genome of ‘*Ca*. P. aurantifolia’ NCHU2014, only one PMU and one transposase gene (*tra5*) were annotated (Figures 2, 6). This may indicate that events of insertion and recombination occurred rarely and, in part, resulted in a small chromosomal size of ‘*Ca*. P. aurantifolia’ NCHU2014. Consistently, only two putative PMUs and one *tra5* gene were annotated in the small genome of ‘*Ca*. P. mali’ AT (Kube et al., 2008). In contrast, multiple putative PMUs and *tra5* genes were annotated in the genomes of ‘*Ca*. P. asteris’ AYWB and OY-M, which have larger chromosomes (Bai et al., 2006; Arashida et al., 2008; Oshima et al., 2013).

A total of 27 genes encoding putative secreted proteins were identified in the genome of ‘*Ca*. P. aurantifolia’ NCHU2014. Among them, 13 were identified as homologs of those present in ‘*Ca*. P. asteris’ AYWB and formed clusters near PMU, except SAP11_E__pWB_ (Figures 2, 7 and Supplementary Table 2). These results are consistent with previous studies regarding that effectors associated with PMUs may be transferred horizontally between different phytoplasmas (Chung et al., 2013; Cho et al., 2019). The SAP homologs with potential biochemical activities of destabilizing plant transcription factors were also identified in ‘*Ca*. P. aurantifolia’ NCHU2014 (Figure 7). However, the sequence identities of those putative effectors between ‘*Ca*. P. aurantifolia’ NCHU2014 (16SrII) and ‘*Ca*. P. asteris’ AYWB (16SrI) are generally low. Among them, SAP11_E__pWB_ shares 36.7% sequence identity with SAP11_A__YWB_, and SAP54_E__pWB_ shares 60.4% sequence identity with SAP54_A__YWB_ (Figure 8A and Supplementary Figure 3). Nevertheless, SAP11_E__pWB_ was characterized with high activity in destabilizing AtTCP12 and AtTCP18, the integrators of branching signals (Figure 8B). The protein sequence of SAP54_E__pWB_ is identical with PHYL1 of ‘*Ca*. P. aurantifolia’ NTU2011 (Supplementary Figure 3), which is demonstrated to induce the degradation of the floral meristem identity protein APETALA1 and floral organ identity proteins SEPALLATA1/2/3/4 in a proteasome-dependent manner (Iwabuchi et al., 2020). These morphological changes of host plants, including increases of branching and young leaves, are expected to improve the fitness of phytoplasmas and their insect vectors, which may facilitate the spread of phytoplasma diseases (Tomkins et al., 2018; Dermastia, 2019).

Increasing evidence suggests that climate change, particularly global warming, is considered to play a role in facilitating the spread of phytoplasma diseases through the population dynamics of insect vectors (Krishnareddy, 2013). Recently, we demonstrated that multiple plant species, including soybean (*Glycine max* L.), mungbean (*Vigna radiata* L.), snake gourd (*Trichosanthes cucumerina* L.), threeflower tickclover (*Desmodium triflorum*), lilac tasselflower (*Emilia sonchifolia*), and *Ixeris Chinensis* could be attacked by 16SrII-V subgroup phytoplasmas in Taiwan (Chen et al., 2021; Chien et al., 2021a,b,c; Wang et al., 2021; Weng et al., 2021). Thus, a better understanding of the 16SrII-V subgroup of phytoplasmas is required to develop effective strategies to combat phytoplasma diseases, which represent an emerging threat to agriculture in Taiwan. In this study, a high-accuracy and complete phytoplasma genome was obtained based on the enrichment of uncultivated phytoplasma cells through immunoprecipitation to accelerate the understanding of pathogenicity of ‘*Ca*. P. aurantifolia’ NCHU2014. Although up to 36% of the reads obtained with the antibody-enrichment procedure were mapped to the phytoplasma genome, reads originating from the plant host still account for a large portion of the total reads. This result likely stems from interactions of phytoplasmas with plant cells, which results in the co-purification of plant cells during the procedure. For further improvement, selecting infected plant samples with high phytoplasma titers may help. However, this is challenging because the number of phytoplasma cells can vary among individual plants propagated from the same infected plant (Christensen et al., 2004). Alternatively, further purification of the antibody used for immunoprecipitation may also improve the enrichment results. However, the polyclonal antibody that specifically recognizes ‘*Ca*. P. aurantifolia’ NCHU2014 might not be suitable against a wide range of phytoplasmas because Imp proteins are highly variable and show low similarities between phytoplasmas even in the same species (Morton et al., 2003; Kakizawa et al., 2006b). Thus, generation of antibodies against individual Imps are necessary to enrich a wide range of phytoplasmas by immunoprecipitation. Moreover, those antibodies could be very useful for phytoplasma examinations during field surveys as Imps are highly abundant proteins in the cell membrane of phytoplasmas.

## Data Availability Statement

The complete genome of ‘*Ca*. P. aurantifolia’ strain NCHU2014 has been deposited under the accession numbers CP040925 (chromosome) and CP040926 (plasmid). This genome sequencing project and the associated raw reads were deposited in the NCBI under BioProject PRJNA294131.

## Author Contributions

CT, Y-YC, C-JW, C-WW, and J-YY performed the experiments. Y-CL, J-RL, LC, and C-HK analyzed the data. CT, Y-CL, C-WW, Y-CC, and J-YY prepared the figures and Supplementary Material. J-YY and C-HK designed the experiments, acquired the funding, wrote the manuscript, and supervised the project. All authors contributed to the article and approved the submitted version.

## Conflict of Interest

The authors declare that the research was conducted in the absence of any commercial or financial relationships that could be construed as a potential conflict of interest.

## Publisher’s Note

All claims expressed in this article are solely those of the authors and do not necessarily represent those of their affiliated organizations, or those of the publisher, the editors and the reviewers. Any product that may be evaluated in this article, or claim that may be made by its manufacturer, is not guaranteed or endorsed by the publisher.

## Supplementary Material

Supplementary Figure 1 Molecular examinations of ‘*Ca*. P. aurantifolia’ NCHU2014-infected periwinkle (*Catharanthus roseus*) and ‘*Ca*. P. luffae’ NCHU2019-infected loofah (*Luffa aegyptiaca*). Genomic DNA samples prepared from healthy (H) and symptomatic (S) plants were examined for the 16S rRNA gene by nested PCR with the universal primers P1/P7 followed by R16F2n/R16R2. A DNA fragment of expected size 1.2 kb was observed only in the symptomatic plants and none in the healthy plants. Arrow indicates the 1.2 kb DNA fragment of phytoplasma 16S rRNA gene.

Supplementary Figure 2 The pairwise genome alignment of ‘*Ca*. P. aurantifolia’ strains NCHU2014 (CP040925) and NTU2011 (NZ_AMWZ01000001.1-13.1). Matches on the same strand and the opposite strand are labeled in red and blue, respectively.

Supplementary Figure 3 Sequence comparison of SAP54/PHYL1 homologs identified in ‘*Ca*. P. asteris’ AYWB (AYWB_224), ‘*Ca*. P. ziziphi’ Jwb-nky (CWO85_00800), ‘*Ca*. P. ziziphi’ Jwb-nky (CWO85_03080), ‘*Ca*. P. aurantifolia’ NCHU2014 (EpWB_v2c2400), ‘*Ca*. P. aurantifolia’ NTU2011 (PnWB_v1c0190), and ‘*Ca*. P. asteris’ OY-M (PAM_049). SAP54/PHYL1 homologs without the signal peptide were aligned by MEGA 7.0 using ClustalW. The sequence alignment was then edited by Jalview software in which identical residues are shaded in blue (upper panel). The color gradient indicates the level of sequence conservation at each position. The sequence identity and sequence similarity between SAP54/PHYL1 homologs are presented on the lower panel.

Supplementary Table 1 Prediction of putative secreted proteins based on SignalP-4.1 and SignalP-5.0.

Supplementary Table 2 List of genes that encode putative secreted proteins. The genes are organized by the homologous gene clusters (HGC) and identified by locus tags. Annotation available from the AYWB or OY-M genomes are provided in the “note” field.
