# Supplementary figures and images for: Accelerating Complete Phytoplasma Genome Assembly by Immunoprecipitation-Based Enrichment and MinION-Based DNA Sequencing for Comparative Analyses
Source: Front Microbiol. 2021 Nov 11;12:766221. doi: 10.3389/fmicb.2021.766221 (PMC8632452; doi:10.3389/fmicb.2021.766221)

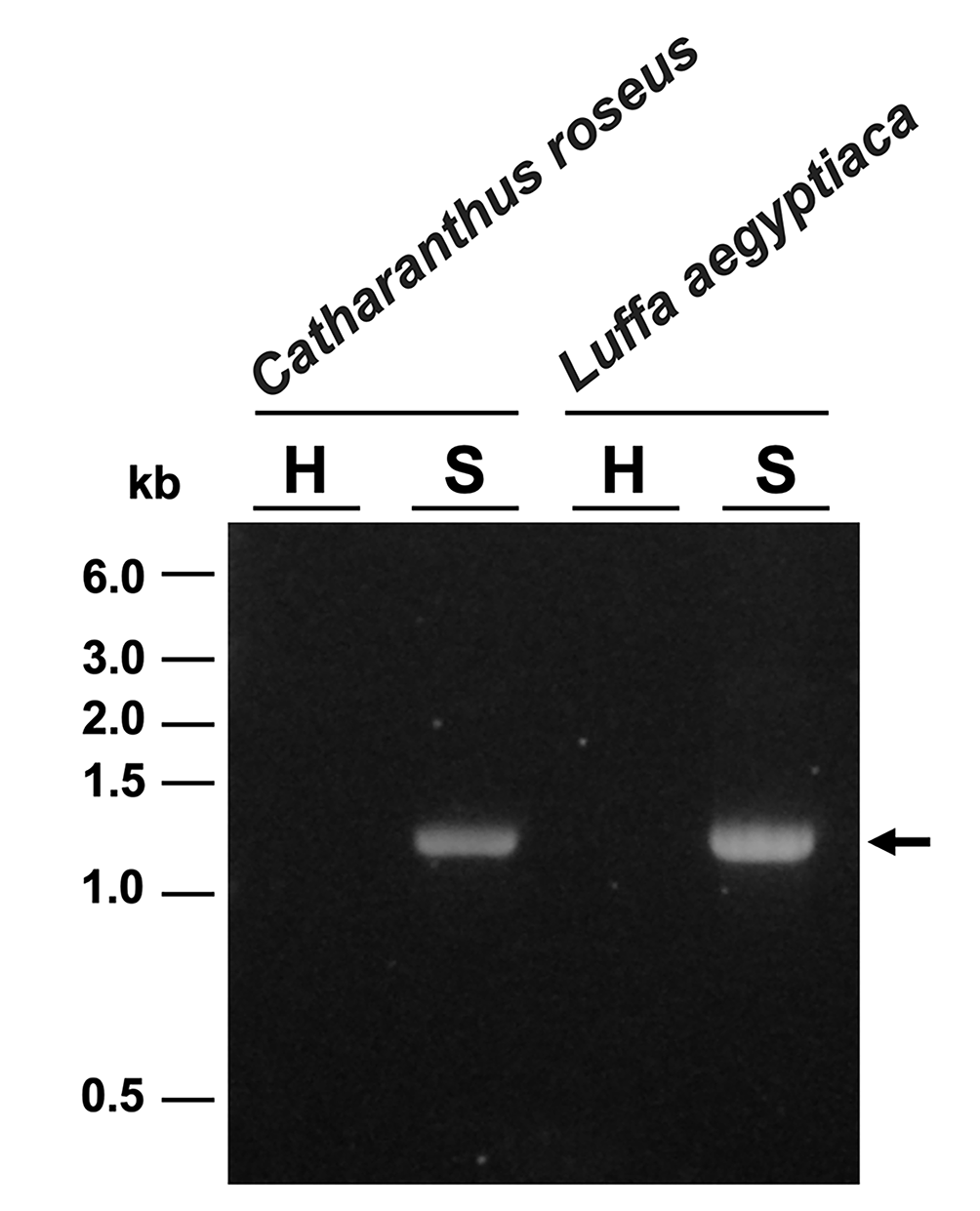

Supplement: Supplementary Figure 1 — Molecular examinations of ‘Ca. P. aurantifolia’ NCHU2014-infected periwinkle (Catharanthus roseus) and ‘Ca. P. luffae’ NCHU2019-infected loofah (Luffa aegyptiaca). Genomic DNA samples prepared from healthy (H) and symptomatic (S) plants were examined for the 16S rRNA gene by nested PCR with the universal primers P1/P7 followed by R16F2n/R16R2. A DNA fragment of expected size 1.2 kb was observed only in the symptomatic plants and none in the healthy plants. Arrow indicates the 1.2 kb DNA fragment of phytoplasma 16S rRNA gene. [file Image_1.TIF]

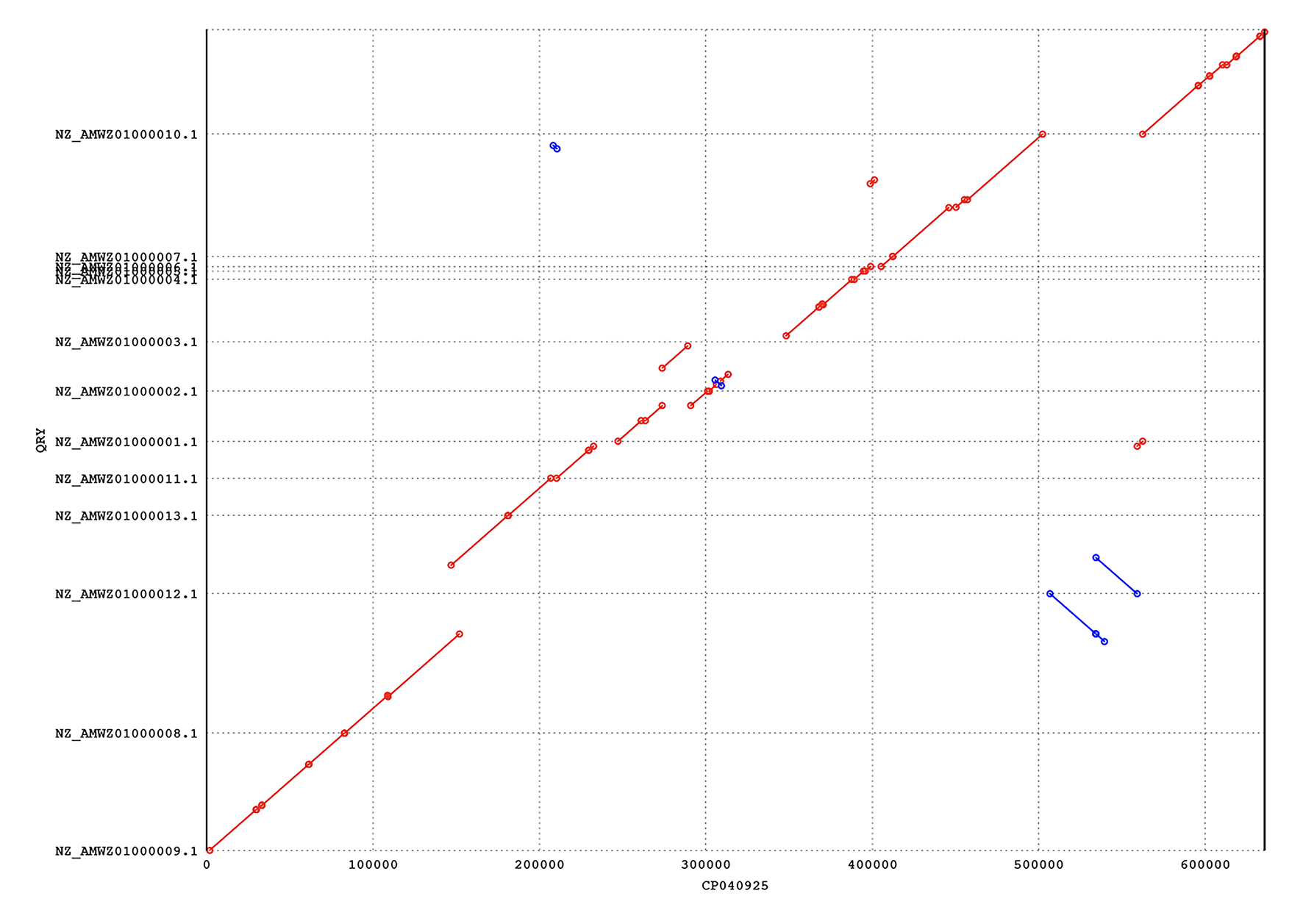

Supplement: Supplementary Figure 2 — The pairwise genome alignment of ‘Ca. P. aurantifolia’ strains NCHU2014 (CP040925) and NTU2011 (NZ_AMWZ01000001.1-13.1). Matches on the same strand and the opposite strand are labeled in red and blue, respectively. [file Image_2.TIF]

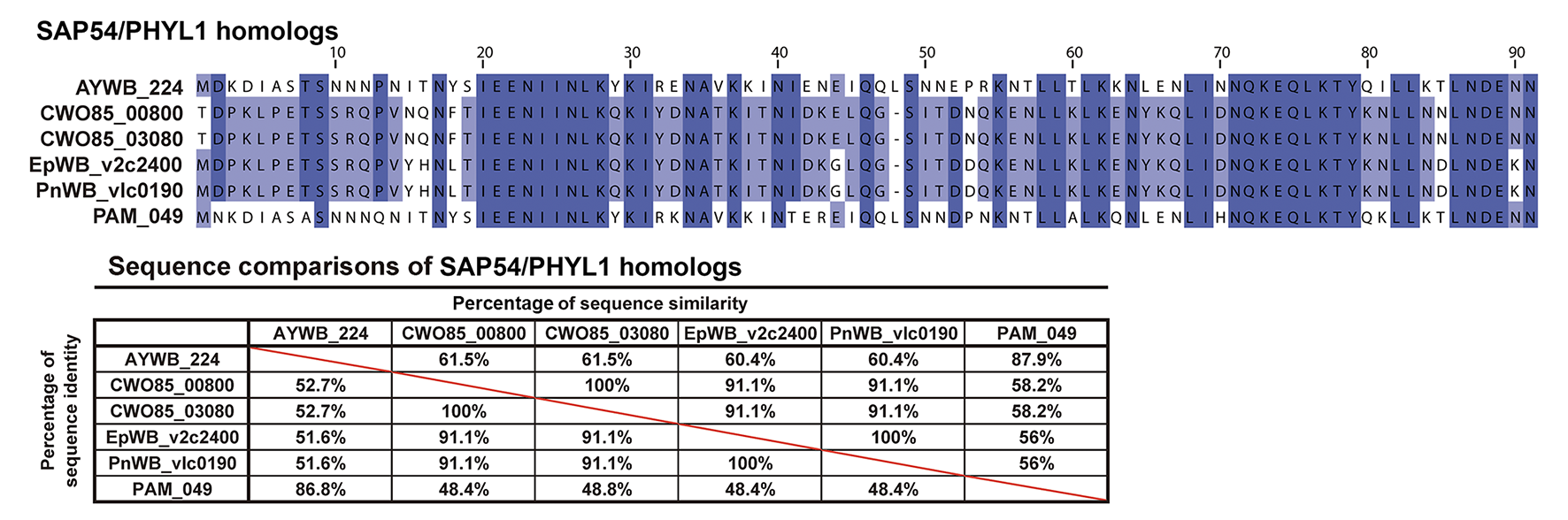

Supplement: Supplementary Figure 3 — Sequence comparison of SAP54/PHYL1 homologs identified in ‘Ca. P. asteris’ AYWB (AYWB_224), ‘Ca. P. ziziphi’ Jwb-nky (CWO85_00800), ‘Ca. P. ziziphi’ Jwb-nky (CWO85_03080), ‘Ca. P. aurantifolia’ NCHU2014 (EpWB_v2c2400), ‘Ca. P. aurantifolia’ NTU2011 (PnWB_v1c0190), and ‘Ca. P. asteris’ OY-M (PAM_049). SAP54/PHYL1 homologs without the signal peptide were aligned by MEGA 7.0 using ClustalW. The sequence alignment was then edited by Jalview software in which identical residues are shaded in blue (upper panel). The color gradient indicates the level of sequence conservation at each position. The sequence identity and sequence similarity between SAP54/PHYL1 homologs are presented on the lower panel. [file Image_3.TIF]
